# Hypersensitivity reaction with multi-organ failure following re-exposure to rifampicin: case report and review of the literature including WHO spontaneous safety reports

**DOI:** 10.1186/s40360-019-0289-7

**Published:** 2019-02-12

**Authors:** Lisa Brockhaus, Yasmin Schmid, Anna C. Rast, Alexandra E. Rätz Bravo, Kathrin Scherer Hofmeier, Anne B. Leuppi-Taegtmeyer

**Affiliations:** 1 Department of Infectious Diseases, University Hospital Basel, Basel, Switzerland; 2 Department of Clinical Pharmacology & Toxicology, University Hospital Basel, Basel, Switzerland; 3 Allergy Unit, Department of Dermatology, University Hospital Basel, Basel, Switzerland; 4 Regional Pharmacovigilance Centre, Department of Clinical Pharmacology & Toxicology, University Hospital Basel, Basel, Switzerland

**Keywords:** Rifampicin, Hypersensitivity reaction, Pneumonitis, Multi-organ failure, Pharmacovigilance

## Abstract

**Background:**

True hypersensitivity reactions to rifampicin are relatively rare, nonetheless severe manifestations mostly involving a single organ have been documented. We report a case of acute multi-organ failure occurring after a medication error with re-exposure to rifampicin.

**Case presentation:**

A 68-year old patient developed acute hypersensitivity pneumonitis, acute renal failure, acute liver failure and haemolytic anemia within hours after a second re-exposure to Rifampicin for the treatment of a hip prosthesis infection with *Staphylococcus epidermidis*. A recent rifampicin exposure 1 week earlier had resulted in a massive rise of CRP levels without organ manifestations. Nine years previously, the patient had developed a multi-organ hypersensitivity reaction 8 days after commencing treatment with rifampicin for pulmonary tuberculosis; and 23 years previously he had received rifampicin without problems. The organ-specific hypersensitivity reactions were largely reversible after withdrawal of rifampicin and treatment with steroids.

A review of the literature and summary of WHO spontaneous safety reports is also given.

**Conclusions:**

Re-exposure to rifampicin in sensitised individuals may cause acute severe hypersensitivity reactions. Due to its indications in the management of mycobacterial and implant-associated infections, rifampicin is a drug which might be given decades apart, which poses a risk that information about previous intolerance is lost.

## Background

Allergic reactions to rifampicin, which is routinely used in the treatment of tuberculosis and implant-associated staphylococcal infections [1], are rarely witnessed in clinical practice [2, 3]. Routine precautions when using rifampicin focus on hepatotoxicity and drug-drug interactions. We report a case of a severe rifampicin hypersensitivity reaction with multi-organ failure occurring at our clinic after accidental rechallenge and discuss the frequency and relevance of this adverse event.

## Case presentation

A 68-year-old Caucasian man (73 kg) was treated for an early postoperative hip prosthesis infection with *Staphylococcus epidermidis* in October 2017. His past medical history included type 2 diabetes, peripheral artery disease, previous coronary artery bypass surgery, a stroke and two episodes of pulmonary tuberculosis, treated in 1994 and 2008.

After surgical debridement of the prosthesis the patient was started on antibiotic therapy with daptomycin. Rifampicin 450 mg twice daily per os (p.o.) was added 12 days postoperatively when the wound was dry, according to treatment concepts of prosthetic joint infections [1]. However, the wound began to discharge again and C-reactive protein (CRP) rose from 90 mg/l to 439 mg/l, and rifampicin was stopped after 3 days of treatment. Common sources of hospital-acquired infections were excluded. Ultrasound examination and joint aspiration did not indicate the presence of an uncontrolled infection. Rifampicin was therefore recommenced a week later.

**Fig. 1:**
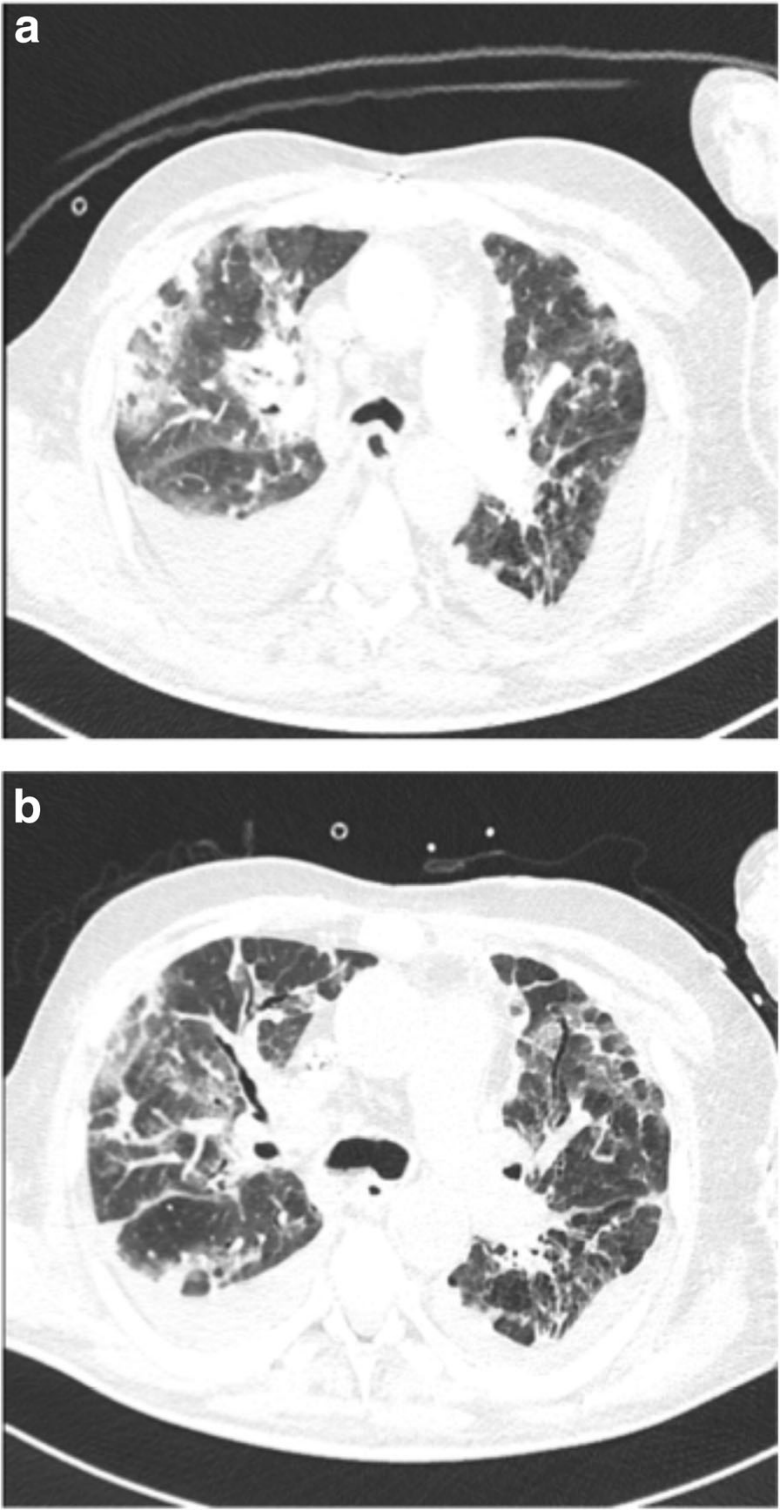
**a**.Ubiquitous ground glass pattern infiltrations; pre-existing post-tuberculosis fibrotic changes in the left upper lobe. **b** Progressive infiltrations, early fibrosis (new traction bronchiectasis anterior left upper lobe)

Two hours after the first rifampicin dose, the patient presented with dyspnea which proved to be rapidly progressive. On clinical examination the patient was hypertensive with a normal heart rate, subfebrile (temperature 37.5 °C), tachypnoeic with an oxygen saturation of 78% on room air, and showed ubiquitous pulmonary crackles. He furthermore developed anuria. A computed tomography (CT) scan of the chest showed ubiquitous ground-glass pattern infiltrations (Fig. 1a). Rifampicin and daptomycin were stopped. The patient was started on hemofiltration for anuric renal failure with marked metabolic acidosis (base excess 18.2, bicarbonate 8.4 mmol/l). His respiratory failure was managed with supplemental oxygen.

Laboratory results during the next few days indicated severe acute liver injury as manifest by massively elevated liver function tests with peak values 2 days after re-exposure to rifampicin (AST 11′115 U/l or 330 times upper limit of normal (ULN), ALT 1′803 U/l or 30 times ULN, LDH 11′883 U/l, total bilirubin 98 μmol/l, spontaneous INR 2.4; previous values all within normal range). Further laboratory abnormalities were eosinophilia (maximum 0.91 G/l), a fall in hemoglobin from 100 g/l to 60 g/l, a positive direct Coombs test, a moderate number of fragmentocytes on the blood film, a urinary sediment with non- glomerular microhematuria without casts, and nephrotic-range proteinuria. The haptoglobin concentration was within the normal range.

Follow-up CT scan of the chest on day 7 after exposure showed progressive ground-glass infiltrations in a “crazy paving” pattern and changes of early fibrosis with new traction bronchiectasis (Fig. 1b), consistent with hypersensitivity pneumonitis. A broncho-alveolar lavage performed on the same day yielded a negative culture, and a cytology specimen showing a moderate cellular infiltration (full cell count 169/ul; ULN 300/ul) of predominantly macrophages (53%) and neutrophil granulocytes (37%). Eosinophilic pneumonia triggered by daptomycin could therefore be excluded.

The patient was started on intravenous steroids (initially methylprednisolone 125 mg once daily (od)) due to the progressive pulmonary changes and daptomycin was re-introduced. Transaminases returned to normal within 1 week. Apart from the temporarily elevated INR, there was no evidence of impaired liver synthetic function. Renal function recovered sufficiently so that hemofiltration could be stopped after 2 weeks, but serum creatinine took 2 months to return to normal range. Pulmonary oxygenation also improved significantly after 2 weeks and a follow up chest CT scan 2 months later no longer showed ground glass infiltrations. Prednisolone was tapered over 2 months as allowed by the clinical course (methylprednisolone 125 mg od for 4 days followed by oral prednisolone 60 mg od for 2 weeks, 40 mg od for 3 weeks, 20 mg od for 3 weeks).

A review of the patient’s tuberculosis treatment records from 9 years previously revealed that management was modified at that time to a rifampicin-free regimen within 8 days of starting treatment due to a suspected rifampicin-hypersensitivity reaction that included kidney failure and hemolytic anemia (Table 1).

**Table 1:** Comparison of reactions in our patient after exposure to rifampicin in 1994, 2008 and 2017

	1994	2008	2017
Rifampicin dose	Rifampicin 700 mg/d p.o. Duration of therapy unknown	Rifampicin 700 mg/d p.o. for 8 days	Rifampicin 900 mg/d p.o. for 2.5 days, stop 6 days, followed by a single oral dose of 450 mg.
Indication for rifampicin	Pulmonary tuberculosis	Pulmonary tuberculosis	Hip prosthesis infection with *S. epidermidis*
Adverse drug reactions (ADR)	None	• Systemic inflammatory reaction • Immune-mediated hemolytic anemia • Mild thrombocytopenia • Interstitial nephritis (biopsy proven) with oligoanuric renal failure requiring CRRT • Transient liver enzyme elevation (AST 10 x ULN, ALT normal)	• CRP-increase from ca. 90 to 450 mg/l after first exposure episode • Systemic inflammatory reaction • Immune-mediated hemolytic anemia • Mild thrombocytopenia • Nephritis with oligoanuric renal failure requiring CRRT • Acute liver injury (AST 330 x ULN, ALT 30 x ULN) • Pneumonitis
Latency time		8 days	< 1 day
Management		• Rifampicin stopped • CRRT	• Rifampicin stopped • Systemic corticosteroids, tapered over 2 months • CRRT
Outcome		Recovery within 2 weeks	Clinical recovery within 2 months
Lymphocyte transformation test		Negative 2 weeks after exposure	Positive 3 weeks after exposure

*CRRT* Continuous renal replacement therapy

A multi-organ hypersensitivity reaction in a patient previously sensitized to rifampicin was therefore diagnosed. Biopsy-confirmation was not performed on account of the suggestive clinical picture, coagulopathy and limited sensitivity after the introduction of steroids. A Rifampicin-specific lymphocyte transformation test (LTT; performed by ADR-AC GmbH, Berne, Switzerland) 3 weeks after exposure was positive even under steroid treatment.

In summary, our patient showed severe acute kidney failure, hypersensitivity pneumonia, acute liver injury and moderate haemolytic anemia after re-exposure to rifampicin.

## Discussion and conclusions

Rifampicin-associated immune-mediated hemolytic reactions as well as acute renal failure and disseminated intravascular coagulation have been described in the literature [4–14], especially in patients who received intermittent dosing regimens. The proposed mechanism is an IgG- and IgM-mediated cytotoxic immune response to I antigens on erythrocytes, platelets, and renal tubular cells [15, 16], with rifampicin acting as the trigger or hapten.

The drug label information for Rifampicin [3] mentions the possible development of a flu-like syndrome that is likely to be immune-mediated if rifampicin is not given on a daily basis or if it is resumed after an interruption. A warning for thrombocytopenia, purpura, dyspnea, bronchospasm, haemolytic anemia, shock, and acute kidney failure without preceding flu-like symptoms in rare cases is included.

Notably, in the first recent exposure to rifampicin during 3 days, a pronounced rise of CRP levels without other explanations was the only apparent reaction. In the second exposure 1 week later, multi-organ failure occurred within hours. Re-processing the case, the patient had previously developed hemolytic anemia and renal failure during rifampicin-containing treatment for pulmonary tuberculosis (Table 1) [4]. A similar case of recurrence of renal failure after re-exposure to rifampicin after 10 years has also been reported in the medical literature [17].

Other medications administered at the time of the multiorgan hypersensitivity reaction – namely pantoprazole, gliclazide, sitagliptin, spironolactone, bisoprolol, atorvastatin, daptomycin, metamizole, lorazepam, trazodone, cholecalciferol and levothyroxine - were assessed in terms of the likelihood that they caused this severe adverse drug reaction, and could be excluded as culprit drugs on the basis of exposure times and known adverse effects.

Pneumonitis has very rarely been associated with rifampicin [18]; the exact underlying mechanism is not known. In two literature cases, the rifampicin-associated pulmonary reactions showed a good response to steroids. Unlike our case, however, they showed a marked increase in lymphocytes in the broncho-alveolar lavage samples [19, 20].

Renal failure due to rifampicin may result from tubular necrosis, interstitial nephritis, or glomerulonephritis [4, 15]. The normal urinary sediment in our patient was not indicative of a specific underlying pathology. Nonetheless, in type II hypersensitivity renal failure results from tubular necrosis and accompanying interstitial nephritis as seen in the patient’s renal biopsy during the first reaction to rifampicin in 2008 [4].

The acute liver failure in our patient might also be explained by a cytotoxic immune response. The laboratory criteria for drug induced liver injury (DILI) were fulfilled (ALT increase >5x ULN and/or ALP increase >2x ULN, or ALT >3x ULN and bilirubin >2x ULN [21]). However, an additional ischemic pathogenesis cannot be excluded considering the rapid normalisation of transaminases and absence of a histological examination. An increase in AST (but not in ALT) to a lesser extent had also been observed during the first hypersensitivity episode in 2008.

Confirmation of immune-mediated organ damage by biopsy was not performed due to risk-benefit considerations. In order to support the clinical diagnosis, a LTT was performed 3 weeks after exposure and was distinctly positive. The LTT measures the proliferation of circulating drug-specific memory T cells in vitro, which proliferate upon drug (i.e. antigen) stimulation, therefore indicating a type IV sensitization. Its reported overall sensitivity is 60–70% and overall specificity at least 85%, depending on the drug. The ideal moment for performing an LTT is 4–8 weeks after the acute phase [22]. Interestingly, an LTT in 2008 2 weeks after exposure was negative, possibly due to too short latency after exposure. We did not perform additional skin tests for type I or type IV hypersensitivity reactions owing to the positive LTT, the temporal relationship to drug intake and the suspected clinical reactions. A further diagnostic test, which might have assisted in confirming our diagnosis of multi-organ hypersensitivity in a patient already sensitised to rifampicin, is the measurement of rifampicin antibodies [23–25]. However, these tests are not routinely available in Switzerland.

The presentation with coombs positive hemolytic anemia in 2008 and in 2017 indicates a type II hypersensitivity, i.e. an antibody-mediated reaction by IgM or IgG targeting membrane-associated antigens.

We therefore hypothesize a recall phenomenon with mixed type II and type IV hypersensitivity reaction.

The case was reported to the pharmacovigilance unit of the Swiss national authority for therapeutic products (Swissmedic). The causality was assessed as “certain” for rifampicin and the development of hypersensitivity-induced multiorgan failure. A “certain” causality can only be conferred to cases of proven positive re-challenge [26], as our patient experienced.

An investigation of the patient’s electronic medical records revealed that the diagnosis of rifampicin-hypersensitivity was documented in all charts until a hospital admission with cellulitis in May 2017, from which point it was “lost”. Information loss was also facilitated by a concurrent transition from paper to electronic patient records. We report this aspect of the case to remind clinicians of the importance of meticulous allergy documentation, ideally in a sustainable fashion including automatic transfer to new case records. Data loss at interfaces during the medication process is a well-known phenomenon [27] and was recently the subject of a patient safety initiative by the Swiss Patient Safety Foundation [28].

We performed an analysis of possible adverse reactions associated with rifampicin reported from 1970 onwards based on the WHO adverse drug reactions global database VigiBase, which contains the largest dataset of postmarketing adverse drug reaction (ADR) reports worldwide. Analysis was performed using the web-based search tool VigiLyze [29], using the MedDRA (Medical Dictionary for Regulatory Activities) coding terms “preferred terms” (PT, term of a single medical concept) or “high level group terms” (HLGT; grouping PTs by anatomy, pathology, physiology, etiology or function) where appropriate. For the liver reaction, the search was also combined with the system organ class (SOC)-term “immune system disorders”, as unlike for the other organ systems, a single term is not given in the dataset. The chosen terms and number of cases are outlined in Table 2. Multiple terms could be reported in one report. It becomes evident that DRESS was reported most frequently, followed by immune reactions involving the liver, hemolysis and kidney disease, while lung involvement was rarely reported.

**Table 2:** Number of rifampicin-associated adverse reactions reported to Uppsala Monitoring Centre (UMC), the Collaboration Centre of the WHO Programme for International Drug Monitoring

	Number of rifampicin-associated adverse reactions in VigiBase (% of total reports) worldwide	Number of rifampicin-associated adverse reactions in VigiBase (% of total reports) in Switzerland
Total rifampicin-associated reports	37,812	192
Drug reactions with eosinophilia and systemic symptoms (PT)	305 (0.8)	9 (5)
Events of hepatic disorders reported with terms of Immune system disorder (SOC)	n.d.*	n.d.*
Hepatitis (PT)	74 (0.2)	3 (2)
Jaundice (PT)	26 (0.1)	–
Acute hepatic failure (PT)	12 (< 0.1)	2 (1)
Hepatic function abnormal (PT)	25 (0.1)	–
Hepatic enzymes increase (incl. ALT, transaminases) (PT)	52 (0.1)	3 (2)
Other hepatic terms reported	Not done	4 (2)
Lower respiratory tract inflammatory and immunological conditions (HLGT)	35 (0.1)	2 (1)
Pneumonitis (PT)	9 (< 0.1)	–
Hemolytic anemia, hemolysis, autoimmune hemolytic anemia (PT)**	187 (0.5)	2 (1)
Tubulointerstitial nephritis, nephritis, allergic nephritis (PT)***	160 (0.4)	6 (3)

* n.d.: in one report, one or more than one PTs may have been reported, therefore the sum is not applicable; ** most cases for haemolytic anaemia; *** most cases for tubulointerstitial nephritis

We found 9 reported cases of pneumonitis, but no explicit cases of hypersensitivity pneumonitis. Out of 104 reports of medication errors associated with rifampicin, two were a positive rechallenge with rifampicin, like our case.

VigiBase data show spontaneously reported, suspected adverse reactions from a variety of sources. No conclusions on incidences can be drawn due to underreporting, reporting bias, coding inconsistencies and lack of exposure data. Furthermore, the information comes from a variety of different sources and the likelihood that the suspected adverse reaction is drug-related is not the same in all cases.

In summary, we report a case of severe hypersensitivity reaction due to re-exposure to rifampicin. The multi-organ involvement (lung, kidney, liver, hemolysis) sets it apart from previously reported cases. Due to rifampicin’s dual indication in the management of mycobacterial and implant-associated infections, it is a drug which might be given decades apart. Physicians should be aware that this poses a risk for information-loss about previous intolerances, strive for meticulous record-keeping and include Rifampicin hypersensitivity reactions in their differential if any unexpected symptoms occur after its introduction.

## Abbreviations

ADR: adverse drug reaction
ALT: alanine transaminase
AST: aspartate transaminase
CRP: c-reactive protein
CRRT: continuous renal replacement therapy
CT: computed tomography
HLGT: high level group term
LTT: lymphocyte transformation test
od: once daily
p.o.: per os
PT: preferred term
SOC: system organ class
ULN: upper limit of normal
